# Percutaneous Method of Management of Simple Bone Cyst

**DOI:** 10.1155/2013/636197

**Published:** 2013-06-02

**Authors:** O. P. Lakhwani

**Affiliations:** ESIC Postgraduate Institute of Medical Sciences and Research, ESI Hospital, Ring road, Basaidarapur, New Delhi 110015, India

## Abstract

*Introduction.* Simple bone cyst or unicameral bone cysts are benign osteolytic lesions seen in metadiaphysis of long bones in growing children. Various treatment modalities with variable outcomes have been described in the literature. The case report illustrates the surgical technique of minimally invasive method of treatment. *Case Study.* A 14-year-old boy was diagnosed as active simple bone cyst proximal humerus with pathological fracture. The patient was treated by minimally invasive percutaneous curettage with titanium elastic nail (TENS) and allogenic bone grafting mixed with bone marrow under image intensifier guidance. *Results.* Pathological fracture was healed and allograft filled in the cavity was well taken up. The patient achieved full range of motion with successful outcome. *Conclusion.* Minimally invasive percutaneous method using elastic intramedullary nail gives benefit of curettage cyst decompression and stabilization of fracture. Allogenic bone graft fills the cavity and healing of lesion by osteointegration. This method may be considered with advantage of minimally invasive technique in treatment of benign cystic lesions of bone, and the level of evidence was therapeutic level V.

## 1. Introduction

Simple bone cysts [1] (SBC) or unicameral bone cysts [1] (UBC) are benign osteolytic cystic lesions of unknown etiology seen at metadiaphyseal region of long bone in growing children. This lesion was first described by Virchow R in 1876 [2]. These lesions are usually asymptomatic and the patient frequently presents with pathological fracture [3] at the time of diagnosis. Various methods of treatment of unicameral bone cysts have been proposed, treatment with open curettage with bone-grafting [4], intralesional injection of steroids [5, 6], autologous bone marrow [7, 8], percutaneous injection of allogenic demineralized bone matrix, and percutaneous curettage and bone grafting [9] with widely variable success rate. A successful treatment should give a higher healing rate, a short time to unite, and no recurrence. 

Minimally invasive technique preserves periosteum, muscles, and blood supply. With curettage cyst decompression and the use of allogenic bone graft, the technique has easy and effective approach. 

## 2. Case Study

A 14-year-old boy presented with chief complaints of pain and swelling over right upper arm and difficulty to move it for 3 days. Mild pain at the right upper arm had been present for around 2 months for which he did not bother. Pain was suddenly increased three days back while he was trying to lift water bucket. On general physical examination, patient had adequate built and nutritional status for his age with no abnormality in general examination. In local examination, tenderness, bony irregularity, and crepitus were present over right proximal humerus. Range of motion was painfully at the right shoulder. Plain radiographs of right upper arm and shoulder revealed central multiloculated lytic lesion with well-defined margins at proximal metadiaphysis with transverse fracture line running through lytic lesion with no new bone formation or parosteal reaction. Laboratory evaluation including complete blood counts with peripheral smear, ESR, serum electrolyte with calcium and phosphate, serum albumin and globulin, renal function test, hepatic function tests, urinalysis and parathyroid hormone all came out to be within normal limits. As per the criteria defined by Chang et al. [10] ratio of the cyst length to the physeal width, it was 3.75 and located 8 mm from the physeal margin, and with these clinical and radiological findings diagnosis of simple bone cyst with pathological fracture was made and, the patient planned for surgical intervention. 

### 2.1. Surgical Technique

The patient was planned for percutaneous cyst curettage and filling of the cyst cavity with deep freezed gamma irradiated allogenic cancellous bone graft under image intensifier guidance under general anesthesia (Figure 2). 

The patient was taken in supine position under general anaesthesia. A small longitudinal skin incision 1.5 cm is made proximal upper arm in the upper extremity and fenestration was created in outer cortex of proximal humerus with 3.2 mm drill bit percutaneously under the guidance of image intensifier. An infant feeding tube was inserted to aspirate out the contents of the cyst which were sent for tissue analysis, a larger suction catheter was introduced, and copious irrigation of the bone cyst was carried out. Flexible titanium elastic nail of 3 mm passed through curved end to break the septae and curette the cyst wall, serosanguinous material content of cyst aspirated and collected through suction catheter. Tip of the nail was advanced distally in medullary canal to further decompress the cyst. Finally allogenic Morselized cancellous bone graft prepared and mixed with aspirated bone marrow packed into the cyst cavity through the drill sleeve under the guidance of image intensifier till whole of the cyst was visibly filled with bone graft wound was closed with skin suture and stabilized with “U” slab. Biopsy report confirmed the cyst to be simple bone cyst. The patient was followed up at regular interval, at 10-week follow-up plain skiagram and CT scan showed healing of fracture and resolution of lesion and the patient was able to attain full range of motion at both the shoulder and elbow joint (Figure 3). 

## 3. Discussion and Conclusion

Cystic bone lesions in the first two decades of life constitute common cause for pathological fractures. Two most important differential diagnoses of cystic lesions in children include unicameral bone cyst and aneurysmal bone cyst. 

Proposed pathogenesis [1] of simple bone cyst includes increased production inflammatory markers described by Komiya et al. [11], increased level of prostaglandin-E2, interleukin -1, and gelatinase inducing bone resorption, and high intraosseous pressure due to venous obstruction hence; local, steroid and curettage of the cyst lining are commonly used method in treatment. K-wire, cannulated screw [12], and flexible intramedullary nail [13] have also been used for providing continous cyst decompression and to dilute inflammatory markers and assist healing. High rate of recurrence [14] after steroid injection leads to the use of other methods including bone marrow and bone graft and bone substitute substitutes like calcium sulphate [15], hydroxyapatite, and so forth, to fill the cavity. 

**Figure 1:**
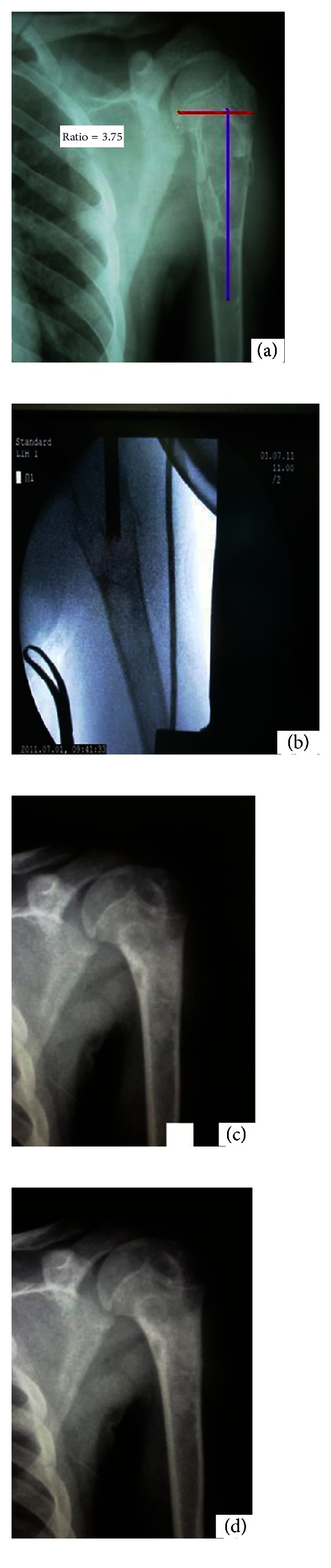
(a) Preoperative skiagram showing lytic lesion with pathological fracture and (b) intraoperative image intensifier skiagram filling of cavity with allogenic bone graft. (c) Follow-up skigam at the 6 month. (d) Follow-up skiagram at most recent follow up at 1 year showed complete healing (more than 95% opacification).

**Figure 2:**
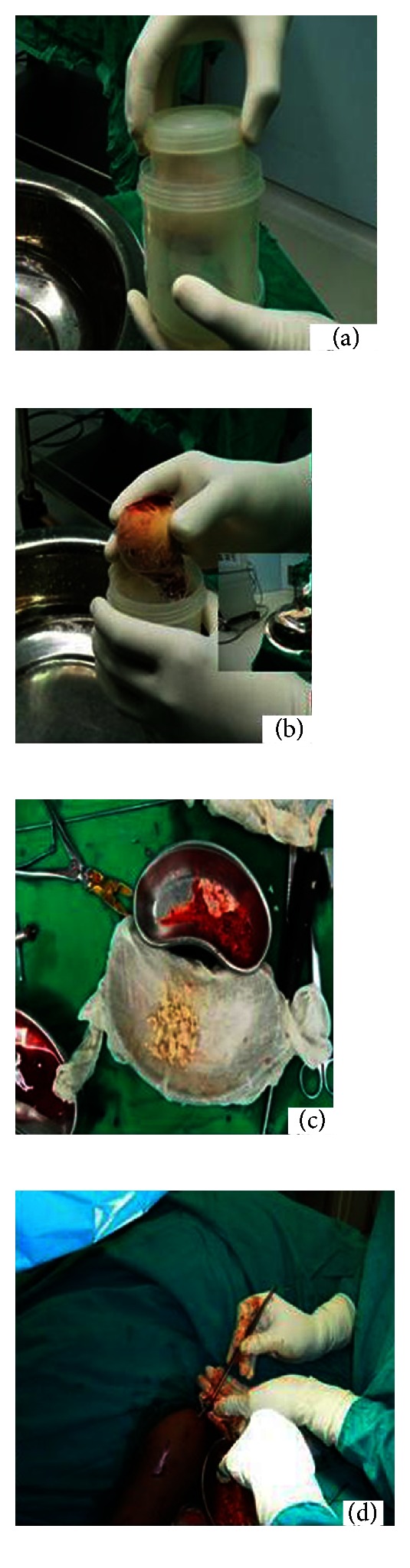
(a) Deep freezed gamma irradiated allogenic bone graft stored in double jar container. (b) Intraoperative picture of graft preparation. (c) Allobone graft before and after mixing with bone marrow. (d) Percutaneous packing of bone graft after curettage and decompression.

**Figure 3:**
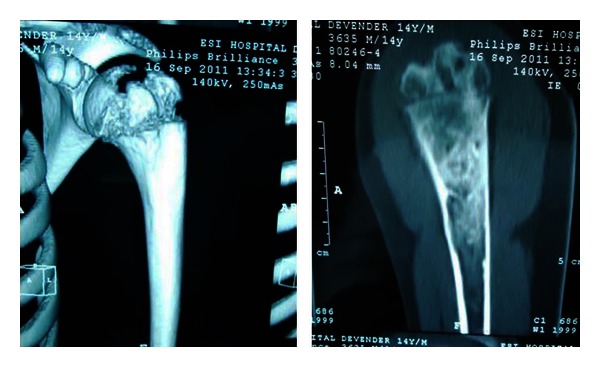
Follow-up CT scan showing healing of lesion.

Open curettage and bone grafting have significant surgical morbidity [16] and recurrence rates of 35 to 45 percent [16, 17]. Similarly, corticosteroids high recurrence rates and need for multiple injections have been reported. Use of allogenic bone graft is comparatively newer method. Spence et al. [18] used the freezed dried cancellous bone allograft to fill the cystic cavity (Figure 1). 

**Figure 4:**
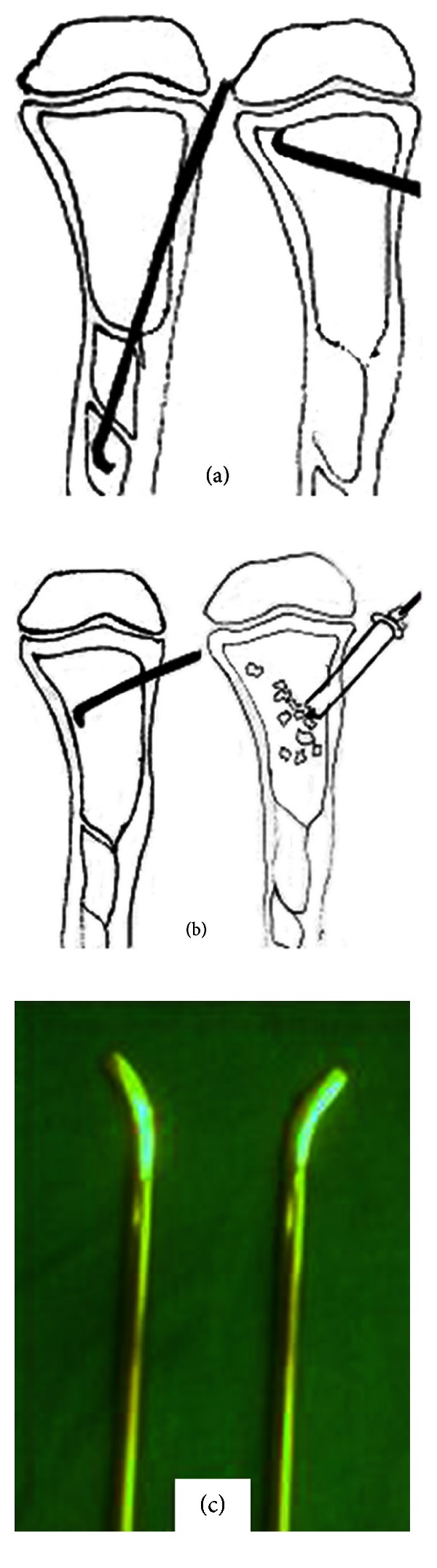
Diagram showing (a) approach for curettage, (b) percutaneous bone grafting, and (c) titanium flexible nail with curved end.

Current surgical technique uses minimally invasive method using the curved end of flexible titanium nail introduced percutaneously for curettage of cyst lining and decompression of cyst (Figure 4). Simultaneously, it can be used for stabilization of fracture. Allogeneic graft obtained after proper screening and gamma sterilization when used with minimally invasive method appears to be the better alternative to the conventional methods of treatments.

In the study by Wright et al. [19], the simple bone cyst trial group comparing rates of healing of simple bone cysts treated with intralesional injections of bone marrow and methylprednisolone acetate has been found to be 42% in methylprednisolone acetate group and 23% in bone marrow injection group. Study by Canavese et al. [20] comparing the healing of unicameral bone cysts by percutaneous curettage, steroid, and autologous bone marrow injections has found that healing of cyst was gretest by mechanical disruption of cyst by percutaneous curettage (70%) compared with bone marrow injection (21%) and methylprednisolone acetate injection (41%). Risk of fracture is important in considering treatment of bone cyst. Pireau et al. [21] used the magnetic resonance scan to calculate the bone cyst index (BCI) described by Kaelin and MacEwen [22] obtained by dividing the cyst area by the diameter of the diaphysis squared s found MRI scan is superior in comparison to plain radiography in assessing fracture risk.

The outcome of the method determined this minimally invasive procedure (healing, as determined radiographically by chang et al. [10]) which includes >95% of opacification of lesion considered as healing. In current case lesion healed by 10 weeks as seen in follow-up skiagram and CT scan with the use of the current technique, the time to achieve healing is very short compared to other methods of treatment and patient regains full functional activities early. 

In summary, percutaneous method under image intensifier guidance using flexible nail for curettage cyst decompression along with allogeneic bone graft is one of the treatment options that may be considered although the method and its effect need further trials, larger series, and comparison with other available treatments to establish its effectiveness.
